# The role of bone modifying agents for secondary osteoporosis prevention and pain control in post-menopausal osteopenic breast cancer patients undergoing adjuvant aromatase inhibitors

**DOI:** 10.3389/fendo.2023.1297950

**Published:** 2023-11-21

**Authors:** Antonio Galvano, Valerio Gristina, Dalila Scaturro, Tancredi Didier Bazan Russo, Sofia Tomasello, Fabio Vitagliani, Federica Carità, Maria La Mantia, Fabio Fulfaro, Viviana Bazan, Giulia Letizia Mauro, Antonio Russo

**Affiliations:** ^1^ Department of Surgical, Oncological and Oral Sciences, University of Palermo, Palermo, Italy; ^2^ Neuromotor and Cognitive Rehabilitation Research Center, Physical and Rehabilitation Medicine Section, Department of Neurosciences, Biomedicine and Movement Sciences, University of Verona, Verona, Italy; ^3^ Department of Biomedical Sciences (BIOMED), University of Catania, Catania, Italy; ^4^ Department of Biomedicine, Neuroscience and Advanced Diagnostics - BIND, University of Palermo, Palermo, Italy

**Keywords:** alendronate, denosumab, secondary osteoporosis, pain control, breast cancer, aromatase inhibitors (AIS)

## Abstract

**Introduction:**

Hormonal therapy (HT) blocks the hormone-mediated growth signal dramatically reducing estrogenic levels with aromatase inhibitors (AIs) becoming a crucial component of the treatment mainstay in patients with early breast cancer (BC). Postmenopausal BC patients receiving HT present with a significant risk of secondary osteoporosis with AIs further reducing estrogen levels and ultimately leading to an accelerated rate of bone resorption and thus decreased bone mineral density (BMD). This was an observational retrospective clinical study that consecutively enrolled early BC patients with osteopenia to compare the impact of alendronate versus denosumab on secondary osteoporosis prevention and pain control.

**Methods:**

We identified two groups of patients treated with denosumab 60 mg by subcutaneous injection once every six months or alendronate 70 mg orally once a week. All the patients underwent a baseline physiatric evaluation (T0) and underwent a follow-up visit after 18 months (T1) together with femoral and vertebral Dual-Energy X-ray Absorptiometry (DEXA) exam evaluating T-Score marks. From September 2015 to December 2019 a total of 50 early (stage I-III) BC patients were considered eligible and consecutively enrolled in our study if they met pre-specified inclusion criteria.

**Results:**

In the entire observed population, the addition of treatment with alendronate or denosumab led to a significant T-score improvement at the lumbar spine level (-1.92 vs -1.52, p=0.03), with a comparable contribution from alendronate (-1.60 vs -1.45, p=0.07) and denosumab (-2.26 vs -1.58, p=0.07). Regarding the femoral region, neither alendronate (-0.98 vs -1.07, p=0.23) nor denosumab (-1.39 vs -1.34, p=0.81) were able to produce any statistically relevant effect. However, concerning pain control, BMAs had a significant impact on reducing NRS scoresin the general population (T1 3.94 vs. baseline 4.32, p=0.007), with a likelyspecific contribution from alendronate (T1 3.52 vs. baseline 3.88, p=0.004) compared to denosumab (T1 4.36 vs baseline 4.76, p=0.12), without any differences in analgesic therapy assumption over time (p=0.93).

**Discussion:**

Both alendronate and denosumab significantly contributed to preventing secondary osteoporosis in early BC patients with low BMD undergoing AIs, mostly at the lumbar spine level. Moreover, alendronate seemed to significantly impact pain control in such patients further supporting alendronate as a cost-effective option in this frail setting, although BMAs particularities should be carefully considered on an individual basis according to specific clinical contexts.

## 1 Introduction

Breast cancer (BC) stands as one of the most prevalent forms of cancer among women worldwide with approximately 80% of all cases presenting with hormone receptor positivity in the post-menopausal setting (1). In such patients, hormonal therapy (HT) blocks the hormone-mediated growth signal dramatically reducing estrogenic levels with aromatase inhibitors (AIs) becoming a crucial component of the treatment mainstay (2). However, alongside the clinical survival benefit, AIs determine short- and long-term sequelae such as so-called cancer treatment-induced bone loss (CTIBL) with consequent fragility fractures (3). As a result, postmenopausal BC patients receiving HT present with a significant risk of secondary osteoporosis with AIs further reducing estrogen levels and ultimately leading to an accelerated rate of bone resorption and thus decreased bone mineral density (BMD) (4). While postmenopausal women typically experience a yearly decrease in BMD of approximately 1%, individuals using AIs can experience a substantial loss of approximately 5% in BMD each year, nearly doubling the risk of secondary osteoporosis and fractures (5). To date, bisphosphonates (BPs, such as alendronate, risedronate, and zoledronate) and denosumab represent the current Food and Drug Administration (FDA)-approved bone modifying agents (BMAs) for treating and preventing osteoporosis in patients with postmenopausal BC undergoing HT (6). Another compelling AI-related side effect is represented by arthralgias and myalgias which generally appear within a few months of starting treatment, often leading to drug substitution or interruption (7, 8). The pharmacological approach for pain control in patients with HT, especially in the adjuvant setting, is very fragmented and to date, there are no reference guidelines. In this frail clinical scenario, no head-to-head comparison between BPs and denosumab in terms of anti-fracture efficacy, BMD loss prevention, and pain improvement has been conducted yet. This retrospective study aims to compare the impact of alendronate versus denosumab on secondary osteoporosis prevention and pain control in women with osteopenia and early BC undergoing adjuvant AIs.

## 2 Materials and methods

### 2.1 Patients

This study was an observational retrospective clinical study that consecutively enrolled early BC patients with osteopenia undergoing BMAs at the Metabolic Bone Diseases Clinic of Physical Medicine and Rehabilitation Department and receiving AIs at the Oncology Department at the University Hospital Paolo Giaccone in Palermo. Patients were defined as eligible if they were at least 18 years old with a histologically or cytologically confirmed diagnosis of estrogen and/or progesterone receptor-positive (ER+ and/or PgR+) surgically resected BC (stage I-III, according to TNM staging system, eighth edition), low BMD (T-score between -1.0 and -2.5), in post-menopausal status, ECOG-PS ≤ 2, receiving at least one AI (letrozole or anastrozole or exemestane). Exclusion criteria were: primary osteoporosis (T-score below -2.5); active bone fracture; bone metastases; severe renal failure; rheumatic diseases; and previous therapy with BPs, denosumab, teriparatide, chemotherapy or bone radiotherapy. The study was carried out according to the declaration of Helsinki after the approval of the local ethical committee. The protocol was fully explained and written informed consent was obtained from each patient before enrolment.

### 2.2 Study design and treatment

To address our hypothesis among the consecutively enrolled patients, we identified two groups of patients treated with denosumab 60 mg by subcutaneous injection once every six months or alendronate 70 mg orally once a week. All the patients underwent a baseline physiatric evaluation (T0) consisting of: anamnestic data collection; physiatric objective exam; definition of anthropometric characteristics of height/weight with Body Mass Index (BMI) calculation; dorso-lumbar x-ray with semi-quantitative Genant method evaluation; femoral and vertebral Dual-Energy X-ray Absorptiometry (DEXA) exam evaluating T-Score marks; first and second level blood and urine tests of bone metabolism. Moreover, the patients filled in the Numerical Rating Scale (NRS), Barthel Index (BI), and the Charlson Comorbidity Index (CCI) for the evaluation of pain intensity, performance in activities of daily living (ADL) and comorbidities, respectively.

All patients received cholecalciferol and calcium supplementation and underwent a follow-up visit after 18 months (T1) from the baseline visit (T0). At T1, the patients underwent a new DEXA exam and a new dorso-lumbar x-ray with semi-quantitative Genant method evaluation to evaluate the BMD and the occurrence of vertebral fractures. DEXA exam was prescribed after 18 months according to the international expert consensus (9, 10).

### 2.3 Statistical analysis

Categorical data are presented as the number observed and group percentage and continuous data are shown as the mean +/- standard deviation. The distribution of patients in the treatment groups (alendronate and denosumab) in consideration of each variable selected at baseline was compared using the heterogeneity chi-square test for categorical variables and the Mann-Whitney test for continuous variables. Differences within groups were explored using the paired parametric or non-parametric tests according to data distribution (paired Student’s t-test or Wilcoxon). One-way ANOVA analysis was performed to investigate differences between treatment groups. To clarify the potential impact of the baseline-recorded clinical variables imbalance in the two treatment groups (alendronate and denosumab) and their potential repercussions on the baseline DEXA’s (femoral and lumbar spine) T-scores and NRS values, we conducted a multivariate linear regression analysis adjusted for age, weight, height, BMI, number of bone fractures, CCI and BI. In the same fashion, to estimate the associations between clinical parameters and NRS improvement probability in the different groups, we performed a logistic regression analysis, producing odd ratios (OR) and their respective 95% confidence intervals (95% CI) as outcomes. A p-value < 0.05 was used as a threshold for statistical significance. All the statistical analyses were performed using SPSS statistics software, version 27 (IBM, Armonk, NY, USA).

## 3 Results

### 3.1 Patients’ characteristics

From September 2015 to December 2019 a total of 50 early (stage I-III) BC patients were considered eligible and consecutively enrolled in our study if they met pre-specified inclusion criteria. The median age was 58 years (range 40-83 years) in the overall population. The median age was 64 years (range 45-83 years) and 56 (range 40-76 years) in the denosumab and alendronate cohorts, respectively. Furthermore, no differences were reported in the mean CCI score between denosumab and alendronate (p=0.27) cohorts. Likewise, similar baseline values were reported for BMI (p=0.6), weight (p=0.5), and analgesics consumption (p=0.76), in both cohorts. Patients in the alendronate cohort were significantly taller than denosumab (p=0.02), with a lesser positive fracture history (p<0.001) and a higher BI (p=0.03). Finally, no significant differences for NRS (T0/NRS; p=0.1) and baseline lumbar and femoral DEXA T-score (T0/T-score;0000 p=0.06) were registered between groups (**Table 1**).

**Table 1:** Baseline demographic and clinical characteristics of the enrolled patients.

	Alendronate (n=25)	Denosumab (n=25)	p-value
Mean (+/-SD or %)			
Age	56 (+/- 10.7)	64 (+/- 11.9)	**0.02**
Weight	69 (+/-11.2)	67 (+/- 15.2)	0.5
Height	1.65 (+/- 0.08)	1.6 (+/- 0.07)	**0.02**
BMI	25.5 (+/-3.7)	26.3 (+/-7.6)	0.6
Analgesics (yes/no)	8/17 (47%)	7/18 (38.9)	0.76
Fractures history	0.36 (+/- 0.7)	2.01 (+/-1.8)	**<0.001**
Basal femoral T-score	1.07 (+/- 1.2)	-1.35 (+/- 1.3)	0.44
Basal lumbar spine T-score	-1.60 (+/- 1.1)	-2.26 (+/- 1.2)	0.06
Basal NRS	3.9 (+/- 1.3)	4.8 (+/-2.3)	0.1
Charlson’s Index	2.68 (+/- 0.9)	3.04 (+/- 1.4)	0.27
Barthel’s Index	89.4 (+/-9.8)	82.6 (+/-12.0)	**0.03**

The bold values provided are statistically significant.

### 3.2 Pain evolution according to denosumab or bisphosphonate therapy

After 18 months of follow-up time, we recorded DEXA’s updated values (T1/T-score) and pain evaluation reported by the patients, according to the NRS scale after antiresorptive therapy (T1/NRS). Namely, we initially conducted a within-patient evaluation to study whether each treatment was able to modify the T0/T-score of the femoral neck and lumbar spine, as well as the T0/NRS score after each treatment. Our results showed that, in the entire observed population, the addition of treatment with alendronate or denosumab led to a significant T-score improvement at the lumbar spine level (-1.92 vs -1.52, p=0.03), with a comparable contribution from alendronate (-1.60 vs -1.45, p=0.07) and denosumab (-2.26 vs -1.58, p=0.07). Regarding the femoral region, neither alendronate (-0.98 vs -1.07, p=0.23) nor denosumab (-1.39 vs -1.34, p=0.81) were able to produce any statistically relevant effect. However, concerning pain control, BMAs had a significant impact on reducing NRS scores in the general population (T1 3.94 vs. baseline 4.32, p=0.007), with a likely specific contribution from alendronate (T1 3.52 vs. baseline 3.88, p=0.004) compared to denosumab (T1 4.36 vs baseline 4.76, p=0.12), without any differences in analgesic therapy assumption over time (p=0.93) (**Table 2**). Then, we also conducted a between-patients evaluation to study whether the different treatments resulted in significant changes in terms of femoral and lumbar spine T0/T-scores, as well as T0/NRS, in the two studied cohorts, since these differences were not significant at the study entry, as previously reported (T0/T-score, p=0.06; **Table 1**). Our results showed that neither alendronate nor denosumab, respectively, were able to substantially affect femoral T-score (T1/T-score -0.98 vs baseline -1.39, p=0.19), lumbar spine T-score (T1/T-score -1.45 vs baseline -1.52, p=0.74), or NRS (T1/NRS 4.36 vs baseline -3.52, p=0.11) (**Table 3**).

**Table 2:** Within-patients evaluation of bone mineral density and pain evolution from baseline (T0) to follow-up visit (T1) according to treatment subgroups.

	Overall			Alendronate			Denosumab		
	T0	T1	p-value	T0	T1	p-value	T0	T1	p-value
Femoral T-score	-1.20	-1.18	0.87	-1.07	0.98	0.23	-1.34	-1.39	0.81
Lumbar spine T-score	-1.92	-1.52	0.03	-1.60	-1.45	0.07	-2.26	-1.6	0.07
NRS	4.32	3.94	**0.007**	3.88	3.52	**0.004**	4.76	4.36	0.12

NRS, number rating scale.

The bold values provided are statistically significant.

**Table 3:** Between-patients evaluation of bone mineral density and pain evolution from baseline (T0) to follow-up visit (T1) according to treatment subgroups.

	Alendronate (A) vs Denosumab (D)					
	T0A	T0D	p-value	T1A	T1D	p-value
Femoral T-score	-1.07	-1.35	0.44	-0.98	-1.39	0.19
Lumbar spine T-score	-1.60	-2.26	0.06	-1.45	-1.52	0.74
NRS	4.76	3.88	0.1	4.36	3.52	0.11

NRS, number rating scale.

### 3.3 T-score evolution and pain onset relationship

In our patient cohort, we observed statistically not significant +0.8% and +8.45% mean changes in femoral T-score (p=0.83) following treatment with alendronate or denosumab respectively, without any contribution to bone health improvement in such skeletal site. Similarly, at the lumbar spine level, the addition of an anti-resorptive therapy led to a clinical improvement in T-scores, resulting in -11.78% and -18.4% mean changes with alendronate and denosumab, respectively, however without any on treatment statistically significant differences (p=0.82). Notably, those patients presenting with a T-score improvement at both femoral and lumbar spine levels were 8 out of 25 (32%) for alendronate and 9 out of 25 (36%) for denosumab, while those with a T-score worsening at both skeletal levels were 3 out of 25 (12%) and 7 out of 25 (28%), respectively. At the same time, we registered the T-scores after the 18-month follow-up period (T1/T-score). We recorded mean femoral T1 score values of -1.49 and 0.98 for denosumab and alendronate therapy, respectively. At the lumbar spine site, we detected T1/T-score values of -1.6 and -1.45, and no differences between groups were registered according to the femoral (p=0.19) or lumbar spine (p=0.74) site (**Table 2**).

Further, to find out any clinical variables affecting the pain control according to treatment subgroups at baseline, we performed a logistic regression analysis. We labeled those patients showing an NRS reduction of at least one point over treatment as NRS responders whereas those who exhibited an increase or stability in NRS score as NRS non-responders. The analysis was adjusted for age, height, weight, BMI, improvement in femoral and/or lumbar spine T-score, number of bone fractures, Charlson index, and Barthel index. Our results revealed that a positive fracture history resulted in a higher likelihood of pain control in the alendronate group (OR: 5.60, 95% CI 1.14 – 27.37; p=0.03), whereas no baseline characteristics seemed to influence the pain control in those patients receiving denosumab.

## 4 Discussion

Despite being the most effective and administered hormonal therapy in the post-menopausal BC setting, AIs detrimentally impact bone health eventually leading to secondary osteoporosis which has been regarded as a particularly concerning adverse effect predisposing to a higher risk of fractures and comorbidities (11). In this vein, BPs and denosumab proved to play a crucial role in the management of both metastatic and non-metastatic BC patients, significantly reducing BMD loss and CTIBL while preventing from secondary osteoporosis (12). However, despite several meta-analyses and prospective trials showing the antiresorptive effect of BPs and denosumab in BC patients (13, 14), existing international guidelines do not provide explicit recommendations for the selection of BMAs with only little data focusing on secondary osteoporotic prevention in early-stage BC patients with low BMD. Further, even in the most studied metastatic setting, a wide-spread consensus to support a direct analgesic role for BPs and denosumab is still lacking, although resulting in to delay the bone pain onset (15). In this retrospective study, both alendronate and denosumab demonstrated a significant fracture risk reduction in early BC patients with low BMD undergoing AIs, clinically improving 18-month T-scores mostly at the lumbar spine level. This is consistent with previously published results in BC that, however, included patients with all BMDs extensively focusing on unselected early-stage or advanced patients with bone involvement undergoing HT (16, 17). Despite the absence of direct comparisons, one could argue that denosumab is perceived as a more convenient option than BPs, albeit notably more expensive and lacking a demonstrable impact on overall survival (18). Nonetheless, a recent meta-analysis indirectly comparing denosumab and zoledronate in the same clinical setting did not show any differences in BMD gain between the two treatment groups, consistently with our results (19). Moreover, a larger network meta-analysis underlined that only one-third of BC patients receiving both BPs and adjuvant AIs presented with baseline T-score ≤ -1.0, leading to inadequate statistical power to detect anti-fracture efficacy and further supporting the originality of our findings in this selected population (20, 21).

To avoid any selection bias, our study consecutively recruited a cohort of patients broadly representative of the overall BC population receiving HT, representing the first experience aimed at investigating the role of BMAs for pain control in selected postmenopausal BC patients with low BMD receiving adjuvant HT. Notably, BMAs seemed to significantly mitigate pain onset in BC patients receiving adjuvant AIs, thus impacting quality of life. Namely, in terms of pain relief, we found that alendronate led to an NRS statistically significant improvement compared to denosumab. Despite a slightly less pronounced positive fracture history in the alendronate group (mean 0.36 vs 2.01 in the denosumab group; **Table 1**), it is worth considering that no differences in terms of baseline pain intensity, comorbidities, T-score, and analgesics consumption over time between the two cohorts were observed. Interestingly, such negligible fracture history resulted in a higher chance of pain control in the BP cohort, whereas none of the baseline clinical variables seemed to influence NRS in the denosumab group despite presenting with a relatively greater fracture history. In this vein, these results concurred with previous reports suggesting that BPs might have a direct analgesic effect and with another larger study that, although including 2,046 women with bone metastases only, did not reveal any significant differences favoring denosumab over BPs in terms of pain severity improvement (22–25). Translating these results in terms of secondary osteoporosis prevention and pain control, the cost-efficacy and the rare musculoskeletal adverse events of oral alendronate should be considered in the clinic, especially when compared to the financial burden of denosumab and the non-negligible toxicities of analgesics or nonsteroidal anti-inflammatory drugs (26–29).

Limitations of the study included the retrospective design, the small sample size, and heterogeneity in the follow-up period that, however reflecting a real‐world scenario, may have affected the final overall results, preventing us from deriving general conclusions.

## 5 Conclusions

To date, international guidelines do not provide specific recommendations for BMAs for postmenopausal BC patients receiving adjuvant HT. Our retrospective findings confirmed that both alendronate and denosumab significantly contributed to preventing secondary osteoporosis in early BC patients with low BMD undergoing AIs, mostly at the lumbar spine level. Moreover, alendronate seemed to significantly impact pain control in such patients further supporting alendronate as a cost-effective option in this frail setting, although BMAs particularities should be carefully considered on an individual basis according to specific clinical contexts.

## Data availability statement

The data analyzed in this study is subject to the following licenses/restrictions: Data could be available at reasonable request to the authors. Requests to access these datasets should be directed to antonio.russo@unipa.it.

## Ethics statement

The studies involving humans were approved by Palermo 1 Institutional Ethic Review Board (protocol code 06/2020, 17/06/2020). The studies were conducted in accordance with the local legislation and institutional requirements. The participants provided their written informed consent to participate in this study.

## Author contributions

AG: Conceptualization, Data curation, Methodology, Software, Validation, Visualization, Writing - original draft. VG: Conceptualization, Methodology, Software, Validation, Visualization, Writing - original draft, Writing - review & editing. DS: Conceptualization, Data curation, Investigation, Resources, Visualization, Writing - original draft. TDBR: Investigation, Writing - original draft. ST: Investigation, Writing - review & editing. FV: Investigation, Writing - review & editing. FC: Formal analysis, Writing - review & editing. ML: Visualization, Writing - review & editing. FF: Writing - review & editing. VB: Project administration, Supervision, Writing - review & editing. GM: Project administration, Supervision, Writing - review & editing. AR: Project administration, Supervision, Writing - review & editing.
